# Acromegaly and colorectal cancer risk: emerging roles of the gut microbiota

**DOI:** 10.3389/fendo.2026.1820034

**Published:** 2026-08-13

**Authors:** Giacomo Fabio Antonio Grifoni, Vittoria Favero, Renato Cesare Cozzi, Iacopo Chiodini

**Affiliations:** 1 Department of Clinical Sciences and Community Health, Dipartimento di Eccellenza 2023-2027, University of Milan, Milan, Italy; 2 Unit of Endocrinology, ASST Grande Ospedale Metropolitano Niguarda, Milan, Italy; 3 Department of Medical Biotechnology and Translational Medicine, University of Milan, Milan, Italy

**Keywords:** acromegaly, colorectal cancer, dysbiosis, GH, gut microbiota, IGF-1, risk stratification, screening

## Abstract

Acromegaly is a rare endocrine disorder characterized by chronic growth hormone (GH) hypersecretion, usually due to a pituitary neuroendocrine tumor, leading to persistently elevated insulin-like growth factor 1 (IGF-1) levels. Although improved biochemical control reduces complications, cancer-related mortality remains a growing concern. Patients with acromegaly exhibit an increased prevalence of colorectal neoplasia, likely driven by sustained GH/IGF-1–mediated epithelial proliferation, reduced apoptosis, and activation of pro-tumorigenic signaling pathways. Concurrently, gut microbiota has emerged as a key modulator of intestinal homeostasis and carcinogenesis. Dysbiosis contributes to colorectal cancer development through chronic inflammation, production of genotoxins, disruption of epithelial barrier, and altered microbial metabolite profiles, including reduced short-chain fatty acids (SCFAs). Increasing evidence supports a bidirectional relationship between the gut microbiota and the GH/IGF-1 axis: microbial composition and SCFAs production can influence systemic and local IGF-1 levels, while GH and IGF-1 modulate microbial diversity, intestinal barrier function, and immune responses. Recent studies suggest that acromegalic patients display distinct oral and fecal microbial signatures, characterized by reduced alpha diversity, altered Firmicutes/Bacteroidetes ratio, and increased abundance of taxa potentially associated with colorectal carcinogenesis. Although causality has not been established, these alterations may interact with hormonal excess, insulin resistance, and systemic inflammation to create a permissive microenvironment for neoplastic transformation. This review provides an integrated clinical and pathophysiological synthesis of current evidence linking acromegaly, gut microbiota alterations, and colorectal cancer risk. Understanding this complex interplay may refine colorectal cancer risk stratification and open avenues for microbiota-targeted preventive or therapeutic strategies alongside endocrine disease control.

## Introduction

Acromegaly is a rare disease characterized by chronic hypersecretion of growth hormone (GH), most commonly derived from a neuroendocrine pituitary tumor (PitNET), causing increased release of insulin-like growth factor 1 (IGF-1) (1). In addition to its characteristic clinical manifestation of acral enlargement, acromegaly is associated with multiple systemic complications, including cardiovascular, metabolic, respiratory, osteoarticular, and neoplastic disorders (2). Although cardiovascular involvement has historically been considered the principal determinant of increased mortality in patients with acromegaly (3), mortality attributable to these complications has declined, likely reflecting the greater efficacy of contemporary therapeutic strategies in achieving biochemical control of IGF-1 levels and reducing overall disease burden (4). Conversely, an increase in cancer-related mortality has been reported (5). Among possible oncologic complications, colorectal tumor is one of the most common findings (6–8), as acromegalic patients have been demonstrated to bear higher risk to develop colonic neoplasms (9, 10).

Whether acromegaly is associated with increased colorectal cancer (CRC)–specific mortality compared with the general population remains a matter of debate (11–13), and no definitive answer has yet been reached (14).

In light of the aforementioned evidence, and considering the significantly higher prevalence of polyps and adenocarcinomas in the ascending and transverse colon among patients with acromegaly compared with non-acromegalic individuals, endoscopic screening by means of total colonoscopy should be routinely performed (6, 15).

Another known risk factor in CRC pathogenesis is the alteration in gut microbiota (16). It is also well known that a unique interdependent relationship exists between the GH-IGF1 axis and the gut microbiota. Indeed, the quantitative richness of the microbiota, as well as the presence of certain specific bacteria, appear to correlate directly with IGF-1 levels, and normal IGF-1 levels seem to reduce microbial dysbiosis and immaturity, leading to normalization of the gut microbiome. Finally, GH is thought to influence gut microbial composition and maturation (17).

This review aims to present a clinically focused and pathophysiological integrated synthesis of available evidence on the relationship between acromegaly, gut microbiota alterations, and CRC.

## The gut microbiota: general concepts and clinical relevance

Humans have developed a symbiotic relationship with a wide community of microorganisms, defined as microbiota, which reside in various environments of human body. The gastrointestinal microbiota represents the largest proportion of microbial colonization (18), and is composed mainly of bacteria and, to a lesser extent, fungi, archaea, and viruses (19).

Gut microbiota in healthy adults is predominantly composed of two major phyla, *Firmicutes* and *Bacteroidetes*, with other less-expressed phyla being *Proteobacteria*, *Verrucomicrobia*, *Actinobacteria*, and *Fusobacteria *(20). Although the gut microbiota shows substantial conservation at higher taxonomic levels, its composition in adults varies considerably from one individual to another (21), according to environmental exposure: many factors - such as dietetic habits, physical exercise, obesity, geographical location, smoke, alcohol, environmental pollutants, exposure to drugs (e.g. antibiotics) - have been shown to affect microbial distribution (22, 23). This variability concerns not only the proportional representation of the main phyla, but also the specific genera and species populating each host (24).

Given the remarkable diversity of its microbial populations and their genomic content, the gut microbiome has been linked to multiple host functions, including maintenance of intestinal homeostasis, growth, metabolism and interactions with the immune, endocrine, and nervous systems (25–27).

Under physiological conditions, immune tolerance toward intestinal commensals is maintained by an integrated network involving the intestinal epithelial barrier, innate immune sensing, tolerogenic antigen-presenting cells (particularly intestinal dendritic-cell subsets), microbiota-derived metabolites, and regulatory T-cell responses. Within this network, Foxp3+ regulatory T cells (T-regs) are central mediators of mucosal homeostasis, limiting excessive inflammatory responses to innocuous microbial antigens through anti-inflammatory mechanisms exploiting cytokines IL-10 and TGF-β (28). Both thymus-derived T-regs (tT-regs) and peripherally induced Tregs (pT-regs) contribute to host-microbiota symbiosis; in the colon, pT-reg differentiation and expansion is strongly shaped by the microbiota and its metabolic products (e.g. short-chain fatty acids, SCFAs) that promote extrathymic T-reg generation (29) and support a regulatory mucosal setpoint that, in turn, helps maintain commensal niches and physiologic metabolite balance (30, 31).

At variance, dysbiosis is defined as the replacement of a healthy gut microbiota by a dysfunctional microbial community with disease-promoting potential (32). It can be determined by several factors which can be influenced by many environmental factors, such as smoking, physical inactivity, obesity, low-fiber diet, pet ownership, alcohol intake and antibiotic treatments (22, 33). Moreover, changes in oral microbiota – known as the second largest microbiome in human body – could influence gut microbiota composition and facilitate bowel diseases (34).

Emerging evidence has already shown dysbiosis as a contributing factor in autoimmune, inflammatory and oncologic diseases (35, 36), besides metabolic disorders such as obesity, metabolic syndrome and type 2 diabetes mellitus (37).

Some of the important tasks associated with gut microbiota are operated by microbial metabolites, such as SCFAs (i.e. butyrate, propionate and acetate), which are obtained through fermentation of non-digestible dietary fibers, or secondary bile acids. These molecules have been suggested to be strongly associated with immunomodulation and inflammatory responses, as well as with the prevention of cancerous proliferation (38).

Besides SCFAs and bile acids, other metabolites which are generated or transformed by gut microbiota from dietary protein and amino acids can decrease (e.g. tryptophan-metabolites, 3-indolepropionic acid) or increase (e.g. diet-derived methylamines, polyamines, reactive oxygen and nitrogen species) CRC risk and progression, also depending on concentration and tissue niche (39, 40). This influence is exerted by modulating epithelial barrier integrity, DNA damage and genotoxic stress, tumor immunity and inflammation through receptor-mediated signaling and epigenetic reprogramming of immune cells (41). A prominent example of microbiota-driven epigenetic modulation is represented by butyrate-mediated inhibition of histone deacetylases, influencing chromatin accessibility and gene transcription (41, 42).

In summary, dysbiosis can be conceptualized as a functional reorganization of the gut microbiota and a shift in its metabolic output, in which taxonomic changes translate into altered microbial metabolite profiles and host-bacterium signaling. Functionally, dysbiosis is often marked by: (i) reduced saccharolytic fermentation and lower SCFA availability, weakening anti-inflammatory signaling (43); (ii) disrupted microbial bile acid biotransformation, frequently reflected by decreased secondary bile acids and altered bile acid receptor signaling that modulates barrier and immunity (44, 45); and (iii) enrichment of pro-inflammatory-associated functions, promoting barrier dysfunction and immune activation (46).

**Figure 1:**
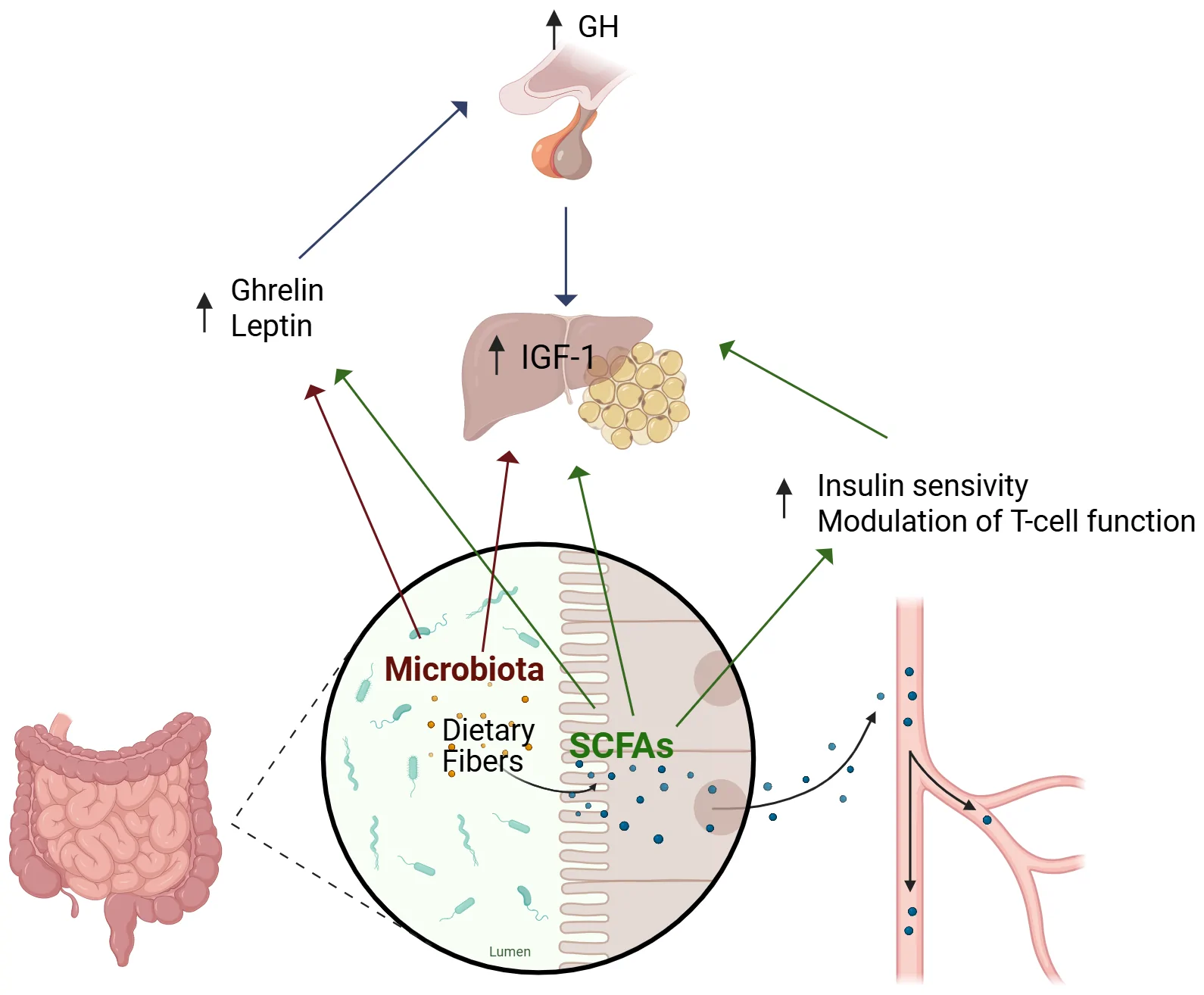
Title: gut microbiota regulation of the GH–IGF-1 axis. Dietary fibers are fermented by the intestinal microbiota into short-chain fatty acids (SCFAs), which increase IGF-1 levels both directly and indirectly. Indirectly, SCFAs enhance insulin sensitivity and modulate T-cell function in peripheral tissues, while also influencing ghrelin and leptin secretion to stimulate GH release and subsequent hepatic IGF-1 production. The gut microbiota can directly elevate both systemic and locoregional IGF-1 levels, while also modulating ghrelin and leptin secretion to further regulate the GH/IGF-1 axis. Created in BioRender.

Furthermore, butyrate has shown context-dependent, paradoxical effects on colon cancer cells based on their metabolic state. Under low-glucose conditions, butyrate is metabolized as an energy source, promoting cell proliferation; conversely, in high-glucose environments characterized by the Warburg effect, butyrate accumulation triggers the inhibition of histone deacetylases, thereby suppressing proliferation (38, 47) **Figure 1**.

## Gut microbiota and the GH/IGF-1 axis

Over the last decade, increasing evidence has shown a bidirectional link between gut microbiota and IGFs, in particular IGF-1 and, to a lesser extent, IGF-2 (48). Gut microbiota seems to increase both systemic – through liver secretion (49) - and locoregional IGF-1 levels (e.g. in adipose tissue) (50). Among the mechanisms underlying this relationship, SCFAs being released into the bloodstream were proposed as an important indirect mediator. Yan et al. highlighted this link in germ-free mice, both through colonization with pathogen-free gut microbiota and through SCFA supplementation in mice previously treated with antibiotics (51). These interventions led to increased IGF-1 levels.

A possible explanation of the IGF-1 accretion may be that SCFAs strengthen the anabolic action of insulin through increased insulin sensitivity and thus improving glucose homeostasis and regulating lipid metabolism regulation (52–55).

While evidence about these matters is strongest for SCFAs as a group, individual SCFA effects are considered more context-dependent and limited. However, butyrate supplementation was demonstrated to enhance the expression of hepatic IGF-1 mRNA and increase IGF-1 production through epigenetic modulation (51, 56). Moreover, excessive chronic propionate (57) and butyrate intake was linked to increased insulinemia and, thus, insulin resistance, confirming the context- and concentration-dependent nature of SCFAs actions.

Fewer data are available regarding the influence of other metabolites on IGF-1 and on the GH/IGF-1 axis in its entirety (58).

Furthermore, SCFAs support the intestinal mucosal barrier by modulating T cell function, ensuring proper nutrients absorption, indirectly influencing IGF-1 (59, 60).

Fewer evidences about SCFAs effects on GH levels are available, as the former have only been shown to suppress GH content and secretion *in vitro* in bovine and goat anterior pituitary cells (17, 61, 62).

Both microbial direct activity and SCFAs production have also been linked to GH/IGF-1 axis regulation through their influence on peptide hormones, such as ghrelin and leptin (27), which enhance GH secretion.

In a murine study, Queipo-Ortuño et al. (63) reported that increased relative abundances of *Bifidobacterium* and *Lactobacillus* were positively associated with serum leptin concentrations, whereas *Clostridium*, *Bacteroides*, and *Prevotella* showed an inverse correlation with leptin levels, independently of dietary restriction. The authors also observed that circulating ghrelin levels were negatively associated with *Bifidobacterium*, *Lactobacillus*, and the *B. coccoides–Eubacterium rectale* group, while positively correlating with *Bacteroides* and *Prevotella* abundance. Furthermore, Parnell and Reimer (64) demonstrated that prebiotic supplementation in obese individuals with oligofructose, which promotes the growth of *Bifidobacterium* and *Lactobacillus*, was associated with reduced ghrelin secretion.

Besides SCFAs-mediated mechanisms, some specific taxa have been found to directly influence IGF-1 levels in humans. In a double-blind randomized controlled trial in older adults, correlation network analysis identified a positive association between circulating IGF-1 and the gut genus *Ruminococcus (*65). Moreover, in a sex-stratified bidirectional two-sample Mendelian randomization designed study, Zheng et al. reported sex-specific causal signals linking gut microbial taxa and circulating IGF-1: among taxa implicated in IGF-1 increase in men were *Deltaproteobacteria*, *Desulfovibrionales*, *Rikenellaceae*, and the genera *Anaerotruncus*, *Eubacterium eligens group*, *Fusicatenibacter*, *Howardella*, *Senegalimassilia*, *Veillonella*, *Ruminococcaceae UCG005*, and *Roseburia*; in women, taxa included *Bacteroidia/Bacteroidales*, *Clostridiales*, *Alcaligenaceae*, *Streptococcaceae*, *Veillonellaceae*, and the genera *Barnesiella*, *Eubacterium ventriosum group*, *Faecalibacterium*, *Lachnospiraceae UCG001*, *Oscillibacter*, *Ruminiclostridium9*, *Ruminococcus1*, and *Veillonella*. However, none of the bacteria passed the false discovery rate correction. On the other side of the coin, the most robust association was represented by an effect of IGF-1 on order *Actinomycetales* in men (60).

Other studies suggested that *Lactobacillaceae* - particularly *Lactiplantibacillus plantarum –* could be implicated in somatotropic axis function in juvenile growth across animal models. In *Drosophila*, specific *Lactobacilli* strains promote juvenile growth by boosting host insulin/insulin-like peptide signaling under chronic undernutrition, a mechanism linked to improved dietary protein digestion and amino-acid uptake (66). In mice, monoassociation of undernourished germ-free juveniles with selected *L. plantarum* strains is reported to be sufficient to restore peripheral GH sensitivity and enhance hepatic IGF-1 activity, partially rescuing postnatal linear growth (17, 66). In livestock models (e.g., broilers and post-weaning lambs), dietary supplementation with *L. plantarum*-derived postbiotic/probiotic preparations has been associated with increased growth performance together with upregulation of hepatic IGF-1-related readouts, supporting a cross-species role for Lactobacillus-linked modulation of the GH/IGF-1 axis from early life (17, 67).

On the other hand, a 2020 review by Jensen et al. (17) also summarized the findings from other studies on the effects of IGF-1 and GH on gut microbiota composition. IGF-1 appears to increase the *Bacteroidetes*/*Firmicutes* phyla ratio in mice, as well as to influence microbial genera (68). In a murine model of persistent low body weight after post-weaning dietary restriction, Chen et al. showed that IGF-1 administration during nutritional rehabilitation remodeled the gut microbiota, mitigating dysbiotic features independently of diet and determining an increase in *Proteobacteria* and enrichment of the genus *Sutterella *(68).

In addition, IGF-1 levels have been suggested to be also positively correlated with Bacteroidetes in human gut microbiota (69). Likewise, GH alterations have been associated with differences in Bacteroidetes/Firmicutes ratio and shifts in specific bacterial genera. A 2020 study in adult male mice by Jensen et al. highlighted opposite microbial signatures between GH deficiency and excess. The former is characterized by reduced *Proteobacteria/Campylobacterota* and by genus-level changes including decreased *Parasutterella*, *Helicobacter* and *Desulfovibrio*, with increased *Bacteroides* and divergent changes among *Firmicutes* genera (e.g., *Turicibacter*, *Ruminococcaceae*). Conversely, chronic GH excess shows an opposing microbial signature, involving genera such as *Parasutterella*, *Bilophila*, *Lactobacillus*, *Turicibacter*, and *Lachnospiraceae/Ruminococcaceae* groups (70).

Finally, GH and IGF-1 indirectly influence the composition and functionality of the gut microbiota, for example through their proliferative effect on enterocytes and intestinal stem cells, or their catabolic action on carbohydrate and lipid metabolism, which produces metabolites that serve as nutrients for the gut bacterial colony. They also modulate the activity of various immune system cells and Paneth cells, stimulating the latter to secrete antimicrobial peptides - small cationic innate immune peptides (e.g., defensins and RegIII lectins), produced also by intestinal epithelial, that directly inhibit and kill bacteria (71) - and helping maintain mucosal barrier integrity and microbiota homeostasis (17, 60, 72–77).

These findings carry important implications, as they suggest rethinking the traditional view of the GH/IGF-1 axis being driven only by the host into the possibility of a broader scenario which also involves the influence of symbiont partners.

## Gut microbiota alterations in acromegaly

Acromegaly is a particular condition that exacerbates the relationship between the GH/IGF-1 axis and the gut microbiota, exposing patients to a chronic elevation in IGF-1, and potentially leading to significant alterations in the composition of intestinal commensal organisms, with possible pathophysiological implications.

Hacioglu et al. (78) first approached this intertwining in human subjects in a pilot cross-sectional study, which included patients with newly diagnosed acromegaly. In this study, fecal samples from patients and selected healthy controls were compared. The study found a significantly reduced bacterial diversity – defined as alpha diversity – in acromegalic subjects, with a predominance of the phylum *Bacteroidetes*, determining a markedly reduced *Firmicutes/Bacteroidetes* ratio, and a decreased representation of other taxa, such as *Bifidobacterium*, *Oscillospira*, and *Dialister *(78). Particularly, genus *Bacteroides* was reported to represent about half of the prokaryotic population. It’s important to highlight that *Bacteroidetes* presence has been linked in the literature to inflammation and carcinogenesis (79); *Firmicutes* on the other hand showed anti-inflammatory effects (80).

While high diversity of gut microbiota has been found to be a protective factor against various disorders (81, 82), reduced alpha diversity appears to be correlated with various diseases, primarily metabolic disorders (83). Moreover, while acromegaly notably enhances risk of metabolic affections, genera with known SCFAs production (e.g. *Bifidobacterium*) were found to be reduced, thus increasing the likelihood of comorbidities (84).

A subsequent larger case-control study by Sahin et al. (85), confirmed that in acromegaly both oral and fecal microbiota profiles differ significantly from those of healthy controls. Patients with active disease exhibited increased oral microbial alpha diversity and a reduced *Firmicutes/Bacteroidetes* ratio in stool samples, with several compositional patterns correlating positively with circulating IGF-1 levels (85).

Metabolic diseases were also demonstrated to cause dysbiosis and hence could have been a confounding factor in this study, as suggested by the authors themselves (78, 85). However, in Sahin’s study a decreased *Firmicutes/Bacteroidetes* ratio was observed despite the absence of concomitant diseases such as diabetes. The effect of this shift on pathogenesis, severity, and response to treatment of the disease is unclear. Furthermore, it remains unclear whether disruption of this ratio plays a causal role in the disease or instead represents a consequence of the pathological condition.

On the other hand, Babayeva et al. (86) reported no correlations between serum IGF-1 and any genera. In this study, alpha diversity was not significantly different between patients and healthy controls, unlike beta diversity (defined as the degree of dissimilarity in microbial community composition across different samples), suggesting that microbial differences between patients with acromegaly and healthy controls are mainly attributable to compositional remodeling, independently of total microbial abundance.

Other studies compared the changes of gut microbiota before and after surgery in patients with GH-secreting PitNETs. A positive correlation between *Enterobacter* at genus and species level and preoperative IGF-1 values, ratio of change in fasting GH and ratio of change in nadir GH levels was found in GH-secreting PitNETs (87), despite the mechanism underlying the interaction between *Enterobacter* and GH/IGF-1 axis remain unclear. A perspective study by Hacioglu et al. (88) gathered longitudinal evidence, showing that microbiota compositions can change significantly with treatment and remission status in acromegaly. Indeed, these authors showed that oral and fecal microbial communities differed across patients who achieved remission through first-line surgery versus those who did not. Only a specific taxon (oral *Succinivibrio*) positively correlated with normalization of serum IGF-1, but patients who instead received second-line use of somatostatin analogues (SSA) exhibited profoundly different oral and gut microbial expression, both in those who achieved remission in such manner and in those who resulted drug-resistant (88).

These findings suggest that certain features of the dysbiotic profile may be at least partially reversible following adequate hormonal control, and baseline microbiota signatures could potentially serve as predictors of therapeutic response.

## Colorectal cancer pathogenesis

### Acromegaly and colorectal cancer pathogenesis

Colorectal cancer (CRC) pathogenesis in the general population is driven by sequential genetic and epigenetic alterations leading from adenoma to carcinoma, frequently involving dysregulation of Wnt/β-catenin signaling, followed by mutations in KRAS and inactivation of TP53, promoting uncontrolled proliferation, resistance to apoptosis, and genomic instability (89, 90). An alternative pathway involving microsate llite instability (MSI) due to mismatch repair defects accounts for a subset of CRCs (91).

In acromegaly persistent exposure to elevated GH and IGF-1 enhances colorectal epithelial proliferation, reduces apoptosis, and promotes a pro-tumorigenic environment. IGF-1 plays a critical role in colorectal tumorigenesis, as IGF-1 activates PI3K/AKT and MAPK pathways, enhancing mitogenesis and inhibiting apoptosis (92, 93). A recent metanalysis confirmed correlations of increases in IGF-1 to upper limit of normal range ratio and insulinemia with higher prevalence of colonic polyps (94).

### Microbiota in colorectal pathogenesis

The gut microbiota has emerged as a causal contributor to colorectal carcinogenesis through multiple, interrelated mechanisms affecting the epithelial barrier, immune responses, and host metabolism. Dysbiosis promotes CRC via chronic inflammation, genotoxin production, metabolite imbalance, and barrier disruption (16, 95). Moreover, some bacterial species were found to potentially contribute to the development of CRC carcinogenesis (96). Microbial-associated molecular patterns, such as lipopolysaccharide, together with bacterial virulence determinants engage host pattern-recognition receptors, including Toll-like receptors, leading to activation of NF-κB signaling cascades. This results in the production of pro-inflammatory cytokines (e.g., IL-6, TNF-α), which perpetuate a chronic inflammatory milieu that favors tumor development by stimulating epithelial proliferation and impairing apoptotic pathways (95, 97).

Lumen (or stool)-associated microbiota (LAM) and mucosa-associated microbiota (MAM) are distinct compartments with partially non-overlapping signatures and functions: MAM/lesion-associated communities are in direct contact with the epithelium and local immunity and may therefore be more relevant to “driver” mechanisms of carcinogenesis, whereas LAM may underrepresent mucosa-adapted and biofilm-forming taxa, while reflecting dietary habits instead. Based on currently available studies it is unknown whether CRC-like compartmentalization concept applies to acromegaly as well (98, 99).

Some gut bacteria produce genotoxic substances; for instance, *entero-toxigenic Bacteroides fragilis* secretes fragilysin, which disrupts E-cadherin and activates β-catenin signaling, while *pks+ Escherichia coli* generates colibactin, directly inducing DNA double-strand breaks in host cells (95, 100). Another key bacterium, *Fusobacterium nucleatum*, is frequently represented in CRC tissues and promotes carcinogenesis through adhesion-mediated activation of β-catenin signaling and suppression of anti-tumor immunity via Fap2 interactions with immune receptors (95, 101).

Cancer-associated microbiomes are often characterized by enrichment of oral bacteria in the colon, such as *Fusobacterium nucleatum*, *Parvimonas micra*, and *Peptostreptococcus stomatis* species, suggesting translocation and establishment of pro-tumorigenic niches (102).

**Figure 2:**
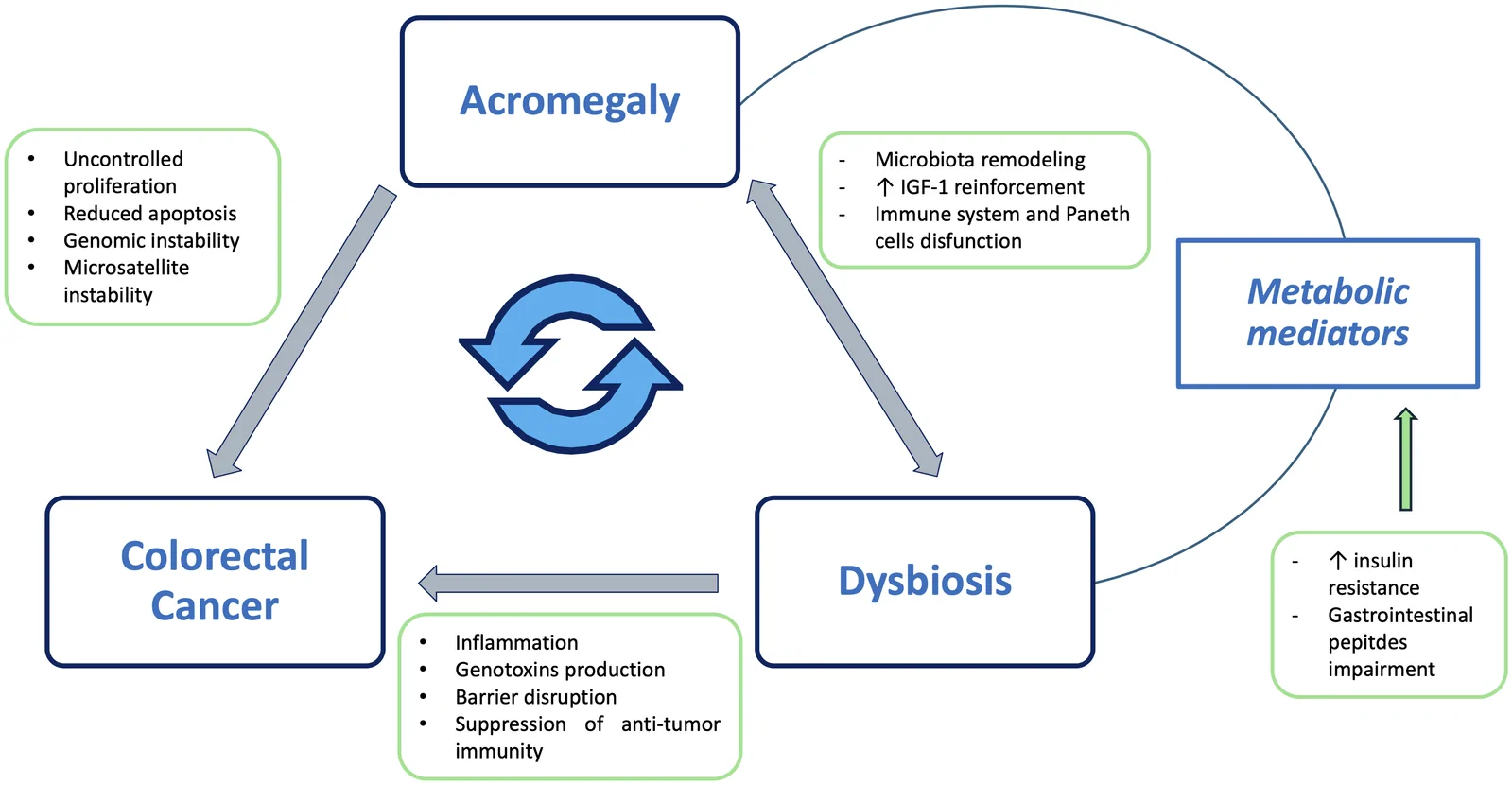
Title: link between acromegaly, gut dysbiosis, and CRC. Key mechanisms linking acromegaly, gut dysbiosis, and CRC risk. Chronic GH/IGF-1 excess and microbial imbalance promote epithelial proliferation, impaired apoptosis, inflammation, genomic instability, and immune dysfunction, fostering a pro-tumorigenic colonic microenvironment.

Conversely, protective microbiota and their metabolites counteract these effects. SCFAs, particularly butyrate, produced by commensals, such as *Bifidobacterium*, *Lactobacilli*, *Roseburia*, and *Clostridium Butyricum*, enhance epithelial barrier integrity, suppress inflammation, promote apoptosis of transformed cells, and modulate immune responses, thereby preventing CRC from developing (97, 103, 104). Depletion of these beneficial taxa and reduced SCFA production are consistent features of CRC-associated dysbiosis (103, 104) **Figure 2**.

## The gut microbiota as a modifier of colorectal risk in acromegaly

Given the increased incidence of CRC reported in acromegaly cohorts in meta-analyses (standardized incidence ratio = 1.95 for colorectal and anal cancers) (98, 105), microbiota alterations should also be taken into consideration during screening and management of acromegalic complications.

Previous studies have already demonstrated increased representation of opportunistic taxa linked with CRC onset, such as *Bacteroides*, *Enterobacter* and *Escherichia* coli (85, 87, 99, 106).

Lin et al. found higher abundance of *Enterobacter*, *Oscillibacter* and *Alistipes* species in acromegalic patients (87), and *Enterobacter* in particular resulted to be strongly associated with GH/IGF-1 axis activity. *Alistipes* was found to promote right CRC tumorigenesis via IL-6/STAT3 pathway, although being a relatively newly isolated bacterium (107). In a study by Parker et al. (107) the manipulation of the tumor microenvironment was suggested as a possible treatment, for example by inducing tumor necrosis factor through tumor-associated myeloid cells, leading to tumor elimination.

Alterations in oral and gut microbiota were also uncovered in patients with colorectal pre-cancerous polyps (108), whose risk is also elevated in acromegaly (109). In another study by Hacioglu and coauthors, acromegalic patients who developed colorectal adenomas exhibited lower expression of *Coprococcus* and *Akkermansia muciniphila* in fecal microbiota (88). The latter has been studied in various contexts and displayed a controversial role towards CRC development, acting in both protective and harmful ways depending on microbial environment, host immune status, dosage and experimental conditions (110).

Such ambivalent relationship with CRC has been attributed especially to a still underexplored strain-level heterogeneity - which may contribute to divergent outcomes - and to consortium-dependency, as its impact can change according to the surrounding microbial community (110). A recent review by Soheilipour et al. highlighted that many CRC papers rely on strain *ATCC BAA-835 (*111), but there is no strong evidence on head-to-head comparisons among different strains.

From a metabolic standpoint, GH excess in acromegaly disrupts insulin signaling, enhances lipolysis, and stimulates the production of pro-inflammatory cytokines in adipose tissue, exacerbating insulin resistance and promoting a state of low-grade systemic inflammation. This altered metabolic environment is recognized as a contributor to colorectal tumorigenesis, as both hyperinsulinemia and insulin resistance have been positively associated with an increased burden of hyperplastic polyps and adenomas in patients with acromegaly (75, 112, 113).

Additionally, definitive mechanistic evidence directly linking gut dysbiosis to impaired insulin sensitivity in acromegaly remains limited. The literature supports the concept that the gut microbiota alterations may amplify systemic inflammation and insulin resistance, thereby sustaining a loop that could promote colorectal cancer pathogenesis in acromegaly (17, 95).

Finally, in acromegaly the intestinal mucosa is chronically exposed to strong proliferative and anti-apoptotic stimulation (114). Moreover, dysbiosis may lower the threshold for neoplastic initiation and accelerate tumor promotion and progression, since disruption of key regulatory mechanisms, including impairment of intestinal barrier integrity, defective immune surveillance, and loss of microbial homeostasis, has been associated with microbially driven carcinogenesis (16, 115). Finally, microbial imbalance alone was demonstrated to be sufficient to promote cancer in animal models (16, 115).

## Clinical implications and future perspectives

According to the latest American Gastroenterological Association (AGA) guidelines (116), colonoscopy remains the recommended first-line screening approach for colorectal cancer in general population, especially in individuals with elevated risk based on family history, although other non-invasive screening options, including fecal immunochemical test, can be evaluated in cases where colonoscopy is declined or not feasible (116).

According to the 2024 acromegaly consensus, no agreement was reached on whether patients should undergo colonoscopy at diagnosis. In contrast, previous consensus recommendations favored performing colonoscopy at diagnosis (117) and adjusting the interval between examinations based on the results of the baseline endoscopic evaluation and IGF-1 levels, with follow-up intervals ranging from 3 to 10 years (118).

Among less invasive screening tests, fecal samples to study gut microbiota composition could provide useful support in acromegalic subjects in the future. Stool microbiome testing is considered the primary method for assessing dysbiosis in clinical and research settings (119). Nowadays, microbiome testing is considered to be easily accessible, as both at-home collection kits and mail-in options are available. Samples are collected with tubes pre-filled with a genome stabilizing solution (119, 120). The cost for stool microbiome testing varies widely depending on the kind of technology being used (i.e. single molecule sequencing technologies or full-length 16S rRNA gene sequencing) and on the depth of analysis, ranging in the hundreds of USD per sample. However, the progressive decrease in costs, along with dissemination of microbiome knowledge, could encourage greater usage in the future (119, 121). Downsides could mainly consist of patient discomfort with handling stool and the need for precise instructions on sample collection, preservation and shipping (120, 122).

Study of gut microbiota could orient physicians not only towards regulating endoscopic screening timing, which are still highly variable as mentioned above. Furthermore, besides standard surgery and medical treatment through SSA or GH antagonists, it could hint at new possible therapeutic approaches in acromegalic patients with altered microbiota showing incremented representation of carcinogenic bacteria, thus possibly increasing CRC risk.

For instance, prebiotics and especially probiotics are already regularly employed in treatment of many conditions, including inflammatory bowel diseases (IBD) and Helicobacter Pylori infection (123), besides providing benefits also in metabolic impairments (124). These microbiome modulators act by improving barrier function, production of SCFA and bile acids, regulating immune system and by directly interacting with commensal bacteria (123, 124).

A more impactful option could be represented in the future by fecal microbiota transplantation (FMT), which so far has only been recommended by the AGA as therapy to prevent recurrent Clostridium difficile infection in select patients (125). It involves the transfer of processed stool from a healthy donor to a recipient executed via different ways (e.g. colonoscopy, capsules, nasoenteric tube), aiming to restore microbial diversity and revert dysbiosis. Its use in other dysbiosis-associated diseases, such as IBD, irritable bowel syndrome, and metabolic syndrome, is under active investigation, but current guidelines do not recommend FMT for these conditions outside of clinical trials yet (126, 127).

## Conclusions

Acromegaly represents a systemic endocrine disorder in which chronic GH/IGF-1 excess drives a complex network of metabolic, inflammatory, and tissue-level remodeling. Within this complex pathophysiological landscape, the gut microbiota emerges as a potentially relevant but still insufficiently characterized component. Current evidence suggests that individuals with acromegaly may exhibit a distinct microbial signature, and that this altered ecosystem could interact with hormonal excess, insulin resistance, and inflammatory signaling to modulate CRC risk. Rather than being a mere bystander, gut dysbiosis may contribute to self-perpetuating pathogenic circuits that reinforce epithelial proliferation, impair immune surveillance, and sustain a tumor-promoting microenvironment in the colonic mucosa.

Although several mechanistic and clinical aspects remain unresolved, incorporating the gut microbiota into the conceptual framework of acromegaly could provide a more integrated understanding of its long-term complications. This perspective may ultimately refine risk stratification for CRC and support the development of preventive or microbiota-targeted strategies alongside endocrine control.

Nonetheless, important limitations must be considered. Available human studies are limited in size, predominantly cross-sectional, and methodologically heterogeneous, precluding causal inferences. Furthermore, it remains uncertain whether the observed microbial alterations are specific to acromegaly, secondary to metabolic comorbidities, or potentially reversible following effective treatment. The extent to which these microbial shifts could be modified and clinically feasible in this population is also unknown.

Moreover, most of the studies regarding microbiota in acromegalic patients were conducted under previous diagnostic criteria (128), as the latest guidelines were only released in 2024 (129).

Despite these issues, the convergence of preliminary clinical findings with strong biological plausibility underscores the need for further investigation. Future longitudinal studies should determine whether biochemical remission and normalization of GH/IGF-1 levels are associated with partial or complete restoration of microbial eubiosis, and whether such changes correlate with improvements in metabolic, inflammatory, and neoplastic risk markers.

Addressing these questions will be crucial to clarify whether modulation of the gut microbiota may represent not only a biomarker of disease activity but also a therapeutic opportunity in acromegaly.
